# A Multi-Modal Fusion Method Based on Higher-Order Orthogonal Iteration Decomposition

**DOI:** 10.3390/e23101349

**Published:** 2021-10-15

**Authors:** Fen Liu, Jianfeng Chen, Weijie Tan, Chang Cai

**Affiliations:** 1 School of Marine Science and Technology, Northwestern Polytechnical University, Xi’an 710072, China; liufen0223@mail.nwpu.edu.cn (F.L.); caichang@mail.nwpu.edu.cn (C.C.); 2 College of Mathematics and Computer Science, Yan’an University, Yan’an 716000, China; 3 State Key Laboratory of Public Big Data, College of Computer Science and Technology, Guizhou University, Guiyang 550025, China; wjtan@gzu.edu.cn

**Keywords:** multi-modal fusion, tensor, iteration decomposition, dimensionality reduction

## Abstract

Multi-modal fusion can achieve better predictions through the amalgamation of information from different modalities. To improve the performance of accuracy, a method based on Higher-order Orthogonal Iteration Decomposition and Projection (HOIDP) is proposed, in the fusion process, higher-order orthogonal iteration decomposition algorithm and factor matrix projection are used to remove redundant information duplicated inter-modal and produce fewer parameters with minimal information loss. The performance of the proposed method is verified by three different multi-modal datasets. The numerical results validate the accuracy of the performance of the proposed method having 0.4% to 4% improvement in sentiment analysis, 0.3% to 8% improvement in personality trait recognition, and 0.2% to 25% improvement in emotion recognition at three different multi-modal datasets compared with other 5 methods.

## 1. Introduction

The multi-modal fusion technique turns up to be an interesting topic in AI technology fields. It integrates the information in multiple modalities and therefore is expected to perform better prediction than the case using any unimodal information [1]. Nowadays it has been applied in a broad range of applications, such as multimedia event detection [2,3], sentiment analysis [1,4], cross-modal translation [5,6,7], Visual Question Answering (VQA) [8,9], etc.

The multi-modal fusion techniques can be typically divided into three approaches, which are the early fusion [10], the late fusion [11] and the hybrid fusion [12]. The early fusion approach extracts the representation of features from each model and then fuses them at the feature level [10]. This approach is more suitable for sentiment analysis. In contrast, the late fusion approach trains the different models at first and then merges them at the decision level [13]. This approach, however, is good at emotion recognition. To take advantage of these two solutions, the hybrid fusion approach was subsequently proposed [14]. Most of the abovementioned methods use simple and straightforward ways to integrate the information parameters, e.g., by merely concatenating or averaging the multi-modal vectors, which cannot make use of the dedicated interrelationships among the multiple models at all [15].

Recently, by leveraging the tensor product representations, many researchers have geared towards achieving rich dynamic interactions in both intra-modality and inter-modality directly to boost the performance [1,15,16,17,18]. Zadeh [16] proposed a tensor fusion network (TFN) which calculates the interaction between different modalities by the cross-product of tensor. Unfortunately, such representations suffer from an exponential growth in feature dimensions and resulting in high cost training process. To tackle this problem, an efficient decomposition method (LMF) is proposed [17] which leads to low-rank tensor factors and much less computational complexity, meanwhile, preserves the capacity of expressing the interactions of modalities. However, the method is still prone to parametric explosions once the features get too long. Meanwhile, it also ignores the local dynamics of interactions that are crucial to the final prediction [15].

Motivated by this problem, in this paper, we make use of higher-order orthogonal iteration decomposition and projection to our tasks. It also ensures that the local dynamics of interactions are preserved with reasonable computational and memory costs [19,20].

The main contributions of our paper are given below:

(1) A tensor fusion method for multi-modalities prediction is proposed based on the higher-order orthogonal iteration decomposition and projection. It can remove the redundant information of duplicated inter-modal while producing fewer parameters with minimal information loss.
(2) The proposed method can tradeoff the dimensionality reduction ratio and the error rate well. Meanwhile, it guarantees that the new tensor is closest to the original tensor in the case of maximal dimension reduction.
(3) The performance of the proposed method has been verified through the evaluation processes on three common available multi-modal task datasets.

## 2. Relevant Mathematical Notations

To make the following algorithm description neat and clearer, some tensor related notations and operations are given at first:

T: a tensor, denoting a higher-order extension of vectors and matrices in this paper.

$T^{\left(n\right)}$: a n-mode unfolded matrix

∥T∥: the Frobenius norm of a tensor T

$\times_{n}$: the n-mode product of a tensor

⊗: the Kronecker product

**Figure 1:**
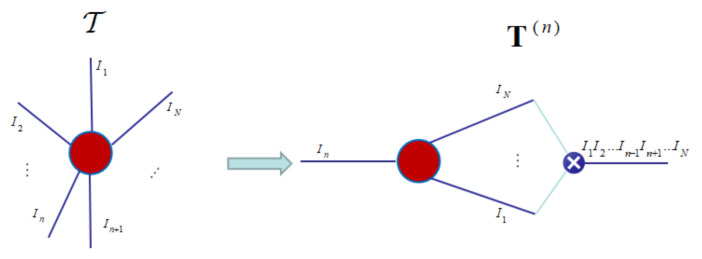
An n-mode unfolding of the *N*-order tensor.

Matricization: also known as unfolding or flattening, is the process of reordering the elements of an N-way array into a matrix. The n-mode matricization of a tensor $T\in R^{I_{1}\times I_{2}\times \ldots I_{N}}$ is denoted by $T^{\left(n\right)}\in R^{I_{n}\times \left(I_{1}I_{2}\cdots I_{n-1}I_{n+1}\ldots I_{N}\right)}$. It arranges the n-mode fibers to be the columns of the resulting matrix [21] as shown in Figure 1:

Tensor Multiplication: The n-mode product of a tensor $T\in R^{I_{1}\times I_{2}\times \ldots I_{N}}$ with a matrix $U^{\left(n\right)}\in R^{J_{n}\times I_{n}}$ is denoted by $T\times {}_{n}U^{\left(n\right)}$ and is of size $I_{1}\times \ldots \times I_{n-1}\times J\times I_{n+1}\times \ldots \times I_{N}$, elementwise, we have

(1) $${\left(T\times {}_{n}U^{\left(n\right)}\right)}_{i_{1},\ldots ,i_{n-1},j,i_{n+1},\ldots ,i_{N}}=\sum_{i_{n}=1}^{I_{n}}t_{i_{1},i_{2},\ldots ,i_{N}}\cdot u_{ji_{n}}$$

Singular Value Decomposition (SVD): A real matrix $A\in R^{m\times m}$ can be expressed as the product

(2) $$A=U\Sigma V^{T}$$

where U and V are orthogonal matrices and Σ is a diagonal matrix.

Tucker’s Tensor Decomposition (Tucker decomposition): Tucker decomposition is higher order SVD. Which approximates tensor $T\in R^{I_{1}\times I_{2}\times \ldots I_{N}}$ with the core tensor $G\in R^{J_{1}\times J_{2}\times \ldots J_{N}}$ and *N* factor matrices $U^{\left(n\right)}\in R^{I_{n}\times J_{n}}\left(n=1,2,\ldots \right)$.

(3) $$T=G\times {}_{1}U^{\left(1\right)}\times {}_{2}U^{\left(2\right)},\ldots ,\times_{N}U^{\left(N\right)}+\varepsilon ,T\in R^{I_{1}\times I_{2}\times .\cdots I_{N}}$$

where ε denotes an arbitrarily small positive real number.

## 3. Methodology

In this section, a multi-modal fusion method based on Higher-order Orthogonal Iteration Decomposition and Projection (HOIDP) is proposed. Similar to many other multi-modal prediction methods, the new method is composed of feature extraction and multi-modal fusion, network model training, and generating prediction task stages. The main contribution of this paper is mainly in the first stage. In another word, it belongs to an early fusion method.

**Figure 2:**
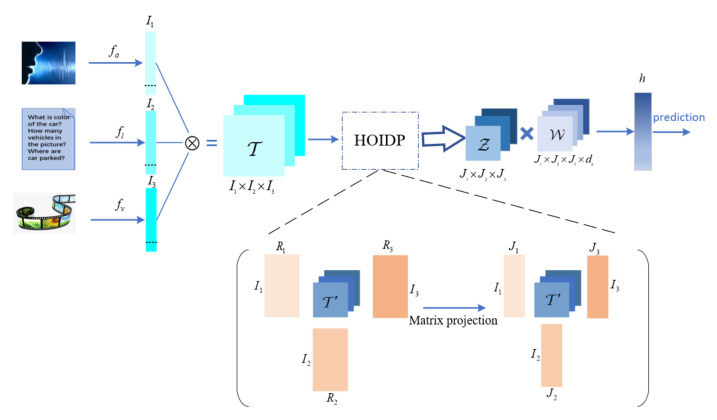
Overview of multi-modal fusion model structure.

As shown in Figure 2, three modalities, i.e., the audio, the text, and the video inputs, are used in our algorithm presentation as well as our following experiments. At first, we obtain the three unimodal representations $I_{1}$, $I_{2}$ and $I_{3}$, which are the outputs of the three sub-embedding networks $f_{a}$, $f_{l}$, and  $f_{v}$ of the audio, the text, and the video input, respectively, with the unimodal feature as their inputs. Secondly, we put these unimodal representations into a tensor T using the Kronecker product and then perform higher-order orthogonal iteration decomposition and projection to get tensor Z. In the end, we put the feature tensor Z into a deep neural network to generate the prediction tasks. The detailed algorithm is introduced in the following subsection.

### 3.1. Multi-Modal Fusion Based on Tensor Representation

Tensor representation is an effective approach for multi-modal fusion. We define *N* modalities as $T_{1},T_{2},\ldots$, and $T_{N}$ which are column vectors of sizes $I_{1},I_{2},\ldots$, and $I_{N}$. We represent a *N*-modal tensor fusion approach by the Kronecker product in mathematical form.

(4) $$T=T_{1}⊗T_{2}⊗,\ldots ,⊗T_{N},T\in R^{I_{1}\times I_{2}\times \ldots I_{N}}$$

Equation (4) can capture multi-modal interactions effectively.

The input tensor $T\in R^{I_{1}\times I_{2}\times \ldots I_{N}}$ then goes through a linear layer f(·) to produce a vector representation *h* as shown in Equation (5).

(5) $$h=f\left(T,W,b\right)=T\cdot W+b,h,b\in R^{d_{h}}$$

where f(·) is a fully connected deep neural network, and W is the weight and *b* is the bias. The weight W is conditioned on the feature tensor T. Since the tensor T is higher dimensional and results increasing computational complexity, a higher-order orthogonal iteration decomposition is proposed in order to improve performance and reduce the data redundancy and parameter complexity in follow subsection.

### 3.2. Higher-Order Orthogonal Iteration Decomposition

We use the Tucker decomposition method to decompose the *N*-order tensor $T\in R^{I_{1}\times I_{2}\times \ldots I_{N}}$ using (3). The solution of the core tensor and factor matrix can be obtained by solving the following optimization problem:

(6) $$\arg\min{∥X-G\times {}_{1}U^{\left(1\right)}\times {}_{2}U^{\left(2\right)},\ldots ,\times {}_{N}U^{\left(n\right)}∥}_{F}^{2}$$

We adopt a higher-order orthogonal iteration decomposition algorithm to solve the above optimization problem to get the core tensor G and the factor matrix $U^{\left(n\right)}$. The core process is described in detail as the following steps:

Step 1: The n-mode unfolded matrix $T^{\left(n\right)}\left(n=1,2,3,\ldots ,N\right)$ of tensor T is calculated, and the singular value decomposition of the n-mode unfolded matrix is carried out respectively to obtain $T^{\left(n\right)}=U^{\left(n\right)}D^{\left(n\right)}V^{{\left(n\right)}^{T}}$, let the left singular value matrix $U^{\left(n\right)}\left(n=1,2,3,\ldots ,N\right)$ be the initial factor matrix $U_{\left(k\right)}^{\left(n\right)}\left(n=1,2,3,\ldots ,N;k=0\right)$.

Step 2: Set k=k+1 and perform the operations: $B_{\left(k\right)}^{\left(n\right)}=T\times_{1}U_{\left(k-1\right)}^{{\left(1\right)}^{T}},\ldots ,\times_{n-1}$$U_{\left(k-1\right)}^{{\left(n-1\right)}^{T}}\times_{n+1}U_{\left(k-1\right)}^{{\left(n+1\right)}^{T}}$, then perform the singular value decomposition of the n-mode unfolded matrix $B_{\left(k\right)}^{\left(n\right)}$ to obtain $B_{\left(k\right)}^{\left(n\right)}={\mathrm{UDV}}^{T}$, and finally let $U_{\left(k\right)}^{\left(n\right)}=U$.

Step 3: Calculate the core tensor of the k-th iteration by using the factor matrix. The core tensor of each iteration is calculated until the convergence condition is satisfied.

Algorithm 1 shows the process.
**Algorithm 1** The higher-order orthogonal iterative decomposition algorithm.**Input**:
the *N*-order tensor T;**Output**:
the core tensor G and the factor matrix $U^{\left(n\right)}$; 1:Initialize the factor matrix $U_{\left(0\right)}^{\left(n\right)}$:Calculated $T^{\left(n\right)}=U^{\left(n\right)}D^{\left(n\right)}V^{{\left(n\right)}^{T}}$ by Equation (2);$U_{\left(k\right)}^{\left(n\right)}\leftarrow U^{\left(n\right)}$; k=0. 2:Update factor matrix:k=k+1;$B_{\left(k\right)}^{\left(n\right)}=T\times_{1}U_{\left(k-1\right)}^{{\left(1\right)}^{T}},\ldots ,\times_{n-1}U_{\left(k-1\right)}^{{\left(n-1\right)}^{T}}\times_{n+1}U_{\left(k-1\right)}^{{\left(n+1\right)}^{T}}$;$B_{\left(k\right)}^{\left(n\right)}={\mathrm{UDV}}^{T}$;$U_{\left(k\right)}^{\left(n\right)}=U$. 3:Compute the core tensor of the k-th iteration: $G_{\left(k\right)}=T\times {}_{1}U_{\left(k\right)}^{{\left(1\right)}^{T}}\times {}_{2}U_{\left(k\right)}^{{\left(2\right)}^{T}}\times ,\ldots ,\times {}_{n}U_{\left(k\right)}^{{\left(n\right)}^{T}}$; 4:**if**${\left|G_{\left(k\right)}-G_{\left(k-1\right)}\right|}_{F}\geq \varepsilon$**then** 5: Go to Step 2; 6:**else** 7: Return G, $U^{\left(n\right)}$; 8:**end if**

### 3.3. Factor Matrix Projection

Through the above algorithm, we obtain the core tensor and matrix factors of the tensor T. Since the factor matrix $U^{\left(n\right)}$ represents the principal components of the tensor in each mode, the column vector of the factor matrix represents the principal components in this mode, and the columns are arranged in descending order according to the energy magnitude - the importance degree of features. Therefore, similar to the singular value decomposition, the factor matrices $U^{\left(n\right)}$ are selected such that they perform projection to the original tensor $T\in R^{I_{1}\times I_{2}\times \ldots I_{N}}$ with the front columns $J_{1},J_{2},\ldots ,J_{N}$ of each factor matrix, as shown in (7).

(7) $$Z=T\times {}_{1}U^{{\left(1\right)}^{T}}\left(1:J_{1},:\right)\times {}_{2}U^{{\left(2\right)}^{T}}\left(1:J_{2},:\right),\ldots ,\times_{N}U^{{\left(N\right)}^{T}}\left(1:J_{N},:\right)$$

We can get an new tensor $Z\in R^{J_{1}\times J_{2}\times \ldots J_{N}}$, which is the order of low dimensions in the new eigenspace compared to the original tensor $T\in R^{I_{1}\times I_{2}\times \ldots I_{N}}$.

Let us replace T with Z in Equation (5) and since weight W conditioned on feature tensor Z, so we also replace W with $\tilde{W}$.

(8) $$h=f\left(T,W,b\right)=T\cdot W+b=Z\cdot \tilde{W}+\tilde{b},h,\tilde{b}\in R^{d_{h}}$$

In practice, we flatten tensors Z and $\tilde{W}$ for reducing the last operation to matrix multiplication.

**Figure 3:**
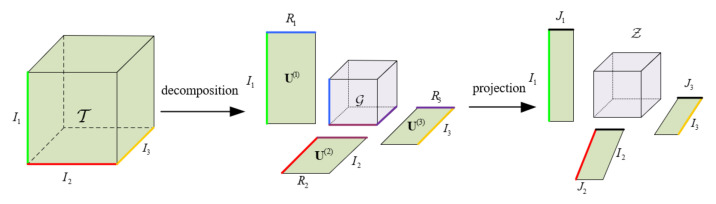
The process of tensor decomposition and projection.

In this paper, we consider the number of modalities to be 3. In Figure 3, tensor $T\in R^{I_{1}\times I_{2}\times I_{3}}$ is decomposed into a core tensor $G\in R^{R_{1}\times R_{2}\times R_{3}}$ and three factor matrices $U^{\left(1\right)}\in R^{I_{1}\times R_{1}}$, $U^{\left(2\right)}\in R^{I_{2}\times R_{2}}$, and $U^{\left(3\right)}\in R^{I_{3}\times R_{3}}$, the three factor matrices are then projected on the front columns $J_{1}$, $J_{2}$, and $J_{3}$. This process can be used for both compression and feature extraction of higher-order data.

## 4. Experimental Methodology

To verify the improvement of the method, we compare our method with DF [22], MARN [23], MFN [24], TEN [16], and LMF [17] in sentiment analysis, personality trait recognition, and emotion recognition at three different multi-modal datasets.

### 4.1. Datasets

Experiments were performed on three multi-modal data sets CMU-MOSI [25], POM [26], and IEMOCAP [27]. Each data set is composed of three modalities: language, video, and audio. The CMU-MOSI includes a collection of 93 comment videos from different film reviews. Multiple opinion clips and emotion annotations consist in each video and are annotated in the range [−3,3], the two thresholds represent highly negative and highly positive respectively. The POM consists of 903 review videos from different movies. Each video has the characteristics of the speaker: self-confidence, enthusiasm, pleasant voice, dominant, credible, vivid, professional, entertaining, introverted, trusting, relaxed, extroverted, thorough, nervous, persuasive, and humorous. IEMOCAP contains 151 videos that are designed to identify emotions displayed in human interactions, such as voice and gesture. The audio-visual data is recorded for approximately 12 h by 10 actors in a two-person conversation. Ten actors were asked to complete three selected scripts with clear emotional content. The dataset contains 9 emotional labels which include anger, happiness, sadness, frustration, and neutral states.

The three datasets include multiple information which has been divided into training, validation, and test sets to evaluate the generalization of the model in this paper. And it is ensured that there are no identical speakers between training sets and test sets. The data split for the three datasets is shown in Table 1.

### 4.2. Multimodal Data Features

Each data set is composed of three modalities, i.e., language, video, and audio. We perform word alignment using P2FA [28] to reach alignment across modalities. The audio and video features can be obtained by calculating the average of feature values in the word time interval [29].

The experiment process of the information is as follows.

- Language: pre-trained Glove word embeddings [30] are used to embed a single word sequence transcribed from video clips into the word vector sequence of spoken text.
- Visual: Facet library is applied for extracting visual features of each frame (sampling at 30 Hz), including head pose, 20 facial action units, 68 facial landmarks, gaze tracking, and HOG features [31].
- Audio: the COVAREP acoustic analysis framework [32] is applied for extracting a set of low-level audio features.

### 4.3. Model Architecture

Three unimodal sub-embedding networks are used to extract representations for each modality [17]. For visual and audio modalities, a simple 2-layer feed-forward neural network is used as a sub-embedding network. And for language, we use a long short-term memory network [33] to extract representations. The model architecture is illustrated in Figure 1.

In this paper, the models are tested using five-fold cross-validation which was proposed by CMU-MOSI. All experiments are performed without the information of speaker identity, while no speaker is repeated in the train and test sets, to make the model universal and independent of speaker information. The hyper-parameters are chosen by using grid search which is based on the performance of the model on the validation set. We trained our model using the Adam optimizer with a learning rate of 0.0003. The subnetworks $f_{a}$, $f_{l}$ and $f_{v}$ are regularized by using dropout on all hidden layers with *p* = 0.15 and L2 norm coefficient as 0.01. The train, validation, and test folds are the same for each of the models. The models are implemented using Pytorch.

### 4.4. Evaluation Metrics

Based on the provided tags, multiple evaluation tasks are performed during our evaluation consisting of multi-category classification and regression. The multi-category classification task is applied to three multi-modal datasets, and the regression task is applied to the POM and CMU-MOSI. For the binary and multi-category classification, the F1 score and the average accuracy (ACC) are used to represent model performance. F1 score can be regarded as a weighted average of precision and recall and can be expressed as

(9) $$F1-score=2\frac{\;\mathrm{precision}\;\cdot \;\mathrm{recall}\;}{\;\mathrm{precision}\;+\;\mathrm{recall}\;}.$$

It has a maximum value of 1 and a minimum value of 0. Similarly, for regression tasks, mean absolute error (MAE) and the correlation (Corr) between prediction and true scores are used to express performance. All these indicators show better performance with the higher values but except for MAE.

## 5. Experimental Results and Discussion

Based on the research questions introduced in Section 3, we present and discuss the results from the experiments in this section.

### 5.1. Comparison with the State-of-the-Art

In the experiment, we compared our model with 5 methods. The Deep Fusion (DF) [22] proposed a concatenation of the deep neural model for each modality followed by a joint neural network. The Multi-attention Recurrent Network (MARN) [23] used a neural component called the Multi-attention block (MAB) which models the interaction between modalities through time and storing them in the Long-short Term Hybrid Memory (LSTM). The Memory Fusion Network (MFN) [24] was proposed for multi-view sequential learning. The Tensor Fusion Network [16] combined each modality into a tensor by computing the outer product. The Low-rank Multi-modal Fusion (LMF) [17] performed the tensor factorization with the same low-rank for multi-modal fusion.

In Table 2, the MAE, Corr, Acc-2, Acc-7, and F1 are presented. The accuracy of the proposed method is marked improvements in CMU-MOSI and POM. It is also marginally better than the LMF method in Happy and Angry recognition.

### 5.2. Computation Accuracy Analysis

The main function of the HOIDP method can achieve the purpose of dimensionality reduction. In this process, the core tensor and factor matrix are obtained by decomposing the original tensor firstly, and then the core tensor with the factor matrix are combined which have been updated by the HOIDP, finally, it forms a projection of the original tensor.

We verified whether the new tensor can replace the original tensor by calculating its error rate. The error rate is measured in norms is shown below:

(10) $$\delta =\frac{{∥T-Z∥}_{F}}{{∥T∥}_{F}}$$

where ${∥T-Z∥}_{F}$ and ${∥T∥}_{F}$ are Frobenius Norms. Since the new tensor is composed of the core tensor and the projection of the updated factor matrix, the dimensionality reduction ratio is defined to measure the similarity between the new and the original tensor as

(11) $$\xi =\frac{N_{nz}\left(G\right)+\sum_{i=1}^{N}N_{nz}\left(U^{\left(i\right)}\left(1:J_{i},:\right)\right)}{N_{nz}\left(T\right)}$$

where $N_{nz}$ is a function that expresses the number of non-zero matrix elements. The dimensionality reduction ratio is generated by calculating the ratio of the non-zero elements in the core tensor and the updated matrix to non-zero elements in the original tensor. This dimension reduction ratio can effectively represent the degree of dimensions reduced.

**Figure 4:**
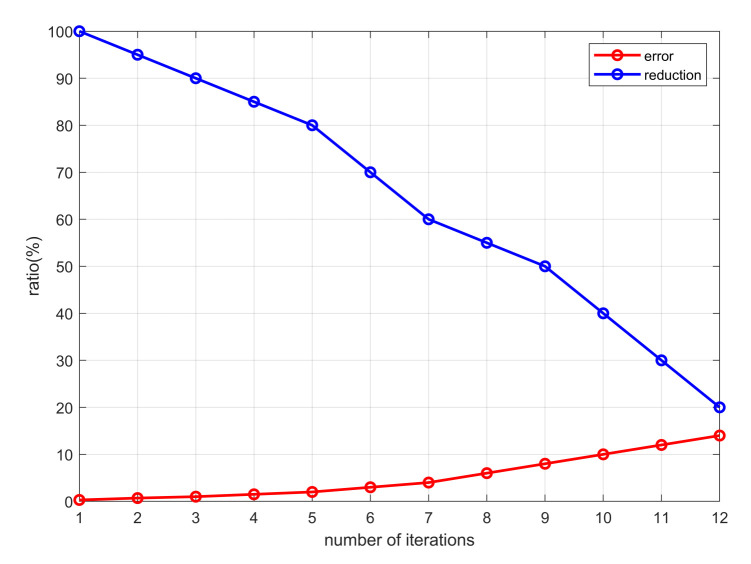
The relationship between dimensionality reduction ratio and error rate.

We use (δ,ξ) to reflect the relationship between the error rate and the dimensionality reduction which is shown in Figure 4. The abscissa is the number of iterations and the ordinate is the ratio value. We set the error rate to 0.3%, 0.7%, 1%, 1.5%, 2%, 3%, 4.1%, 6%, 8.2%, 10%, 11.9% and 14.2% successively. The larger the error rate, the greater difference between the new and the original tensor, and the lower similarity between them.

**Figure 5:**
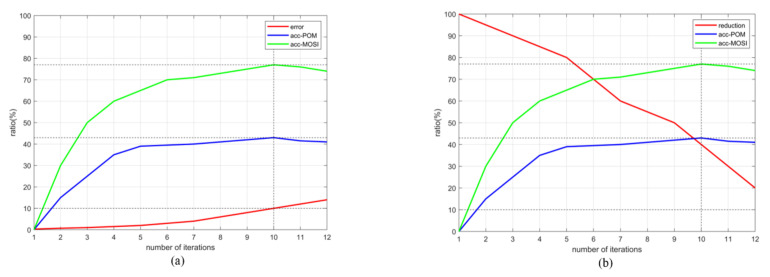
(**a**) The relationship between error rate and ACC. (**b**) The relationship between dimensionality reduction ratio and ACC.

It can be seen from Figure 4 that the lower the dimensionality reduction ratio, the higher is the error rate. It means that we cannot blindly pursue a low dimension in the process of dimensionality reduction. It can achieve a balance between the dimensionality reduction ratio and error rate. In the experimental process, we found that when the number of iterations of tensor decomposition is 10, the error rate is 11.9%, and the dimension reduction ratio is 39.5%. The ACC achieved higher performance on CMU-MOSI and POM data sets as shown in Figure 5, and the prediction results are better when performing the task.

The values of the dimensionality reduction ratio and error rate directly affect the accuracy of feature extraction of multi-modal data, and the evaluation metrics. Therefore, we should ensure that the new tensor is closest to the original tensor in case of maximum dimensionality reduction, and maintain the balance between dimensionality reduction and error according to the different requirements.

Furthermore, to evaluate the computational complexity of HOIDP, we measured the training and test speeds of HOIDP and compared them with TFN and LMF [17] as shown in Table 3. Here we set the dimension reduction error rate to 11.9% and the dimension reduction rate to 39.5% as it can achieve quite a significant increase in performance.

The models are executed in the same environment. The data represents the average frequency value of data point inferences per second (IPS) respectively.

## 6. Conclusions

In this paper, a multi-modal fusion method based on higher-order orthogonal iterative decomposition is proposed, the method can remove the redundant information and leads to fewer parameters with minimal information loss. In addition, we can trade off the dimensionality reduction ratio and the error rate well according to the requirements.

Experiments result show that the method improves the accuracy, the Happy and Angry recognition. It is compared to the other methods and provides the same benefits as the tensor fusion method. It is also immune to a large number of parameters. Furthermore, it can be seen that the HOIDP approach is more efficient and achieves a higher dimensionality reduction effect while maintaining a lower error rate.

## Figures and Tables

**Table 1:** The speaker-independent data splits for training, validation, and test sets [17].

Dataset	CMU-MOSI	IEMOCAP	POM
Level	Segment	Segment	Segment
Train	1284	6373	600
Valid	229	1775	100
Test	686	1807	203

**Table 2:** Results for Sentiment Analysis on CMU-MOSI, personality trait recognition on POM, and emotion recognition on IEMOCAP.

Dataset			CMU-MOSI				POM			IEMOCAP		
Metric	MAE	Corr	Acc-2	F1	Acc-7	MAE	Corr	Acc	F1-Happy	F1-Sad	F1-Angry	F1-Neutral
**DF**	1.143	0.517	72.3	72.1	26.6	0.869	0.144	34.1	81.1	81.2	65.5	44.0
**MARN**	0.967	0.624	77.1	77.0	34.7	-	-	39.5	83.6	81.4	84.5	65.8
**MFN**	0.966	0.632	77.5	77.3	34.3	0.805	0.349	41.7	84.0	82.2	83.7	69.3
**TFN**	0.972	0.634	73.9	73.4	32.2	0.886	0.093	31.6	83.6	82.8	84.3	65.4
**LMF**	0.912	0.668	76.4	75.7	32.8	0.794	0.396	42.7	85.9	85.9	89.1	71.7
**OURS**	0.922	0.663	76.8	75.8	32.0	0.801	0.395	43.1	86.1	85.3	89.2	71.1

**Table 3:** The comparison of training and testing speeds of HOIDP with the TFN and LMF.

Model	Training Speed (IPS)	Testing Speed (IPS)
**TFN**	340.74	1177.17
**LMF**	1132.82	2249.90
**OURS**	1132.42	2253.14

## Data Availability

Not applicable.

## Abbreviations

TFN	Tensor Fusion Network
HOIDP	Higher-order Orthogonal Iteration Decomposition and Projection Fusion
DF	Deep Fusion
MARN	Multi-attention Recurrent Network
MFN	Memory Fusion Network
LMF	Low-rank Multi-modal Fusion
ACC	Accuracy
MAE	Mean Absolute Error
LSTM	Long-sort Term Hybrid Memory
SVD	Singular Value Decomposition
